# Antibacterial Properties of Visible-Light-Responsive Carbon-Containing Titanium Dioxide Photocatalytic Nanoparticles against Anthrax

**DOI:** 10.3390/nano6120237

**Published:** 2016-12-09

**Authors:** Der-Shan Sun, Jyh-Hwa Kau, Hsin-Hsien Huang, Yao-Hsuan Tseng, Wen-Shiang Wu, Hsin-Hou Chang

**Affiliations:** 1 Department of Molecular Biology and Human Genetics, Tzu-Chi University, Hualien 97004, Taiwan; dssun@mail.tcu.edu.tw (D.-S.S.); englishbiology@yahoo.com.tw (W.-S.W.); 2 Institute of Microbiology and Immunology, National Defense Medical Center, Taipei 11490, Taiwan; jhkau@ndmctsgh.edu.tw; 3 Institute of Preventive Medicine, National Defense Medical Center, Taipei 23742, Taiwan; jhhhuang@ndmctsgh.edu.tw; 4 Department of Chemical Engineering, National Taiwan University of Science and Technology, Taipei 10607, Taiwan; tyh@mail.ntust.edu.tw

**Keywords:** anthrax spore, antibacterial agents, TiO_2_, carbon-containing TiO_2_, visible light responsive photocatalyst

## Abstract

The bactericidal activity of conventional titanium dioxide (TiO_2_) photocatalyst is effective only on irradiation by ultraviolet light, which restricts the applications of TiO_2_ for use in living environments. Recently, carbon-containing TiO_2_ nanoparticles [TiO_2_(C) NP] were found to be a visible-light-responsive photocatalyst (VLRP), which displayed significantly enhanced antibacterial properties under visible light illumination. However, whether TiO_2_(C) NPs exert antibacterial properties against *Bacillus anthracis* remains elusive. Here, we evaluated these VLRP NPs in the reduction of anthrax-induced pathogenesis. Bacteria-killing experiments indicated that a significantly higher proportion (40%–60%) of all tested *Bacillus* species, including *B. subtilis*, *B. cereus*, *B. thuringiensis*, and *B. anthracis*, were considerably eliminated by TiO_2_(C) NPs. Toxin inactivation analysis further suggested that the TiO_2_(C) NPs efficiently detoxify approximately 90% of tested anthrax lethal toxin, a major virulence factor of anthrax. Notably, macrophage clearance experiments further suggested that, even under suboptimal conditions without considerable bacterial killing, the TiO_2_(C) NP-mediated photocatalysis still exhibited antibacterial properties through the reduction of bacterial resistance against macrophage killing. Our results collectively suggested that TiO_2_(C) NP is a conceptually feasible anti-anthrax material, and the relevant technologies described herein may be useful in the development of new strategies against anthrax.

## 1. Introduction

Anthrax is a life-threatening infectious disease that spreads through contact with spores of the Gram-positive bacterium *Bacillus anthracis* through skin contact (generally with infected animal products), inhalation, or ingestion [1]. Approximately 2000 to 20,000 cases occur worldwide annually [2], mostly in Africa and central and south Asia [3]. Anthrax spores have been developed as a biological weapon by several countries [4,5,6]. The 2001 US anthrax letter attacks further evidenced an emerging terrorist threat, leading to renewed attention to the importance of prophylaxis, prevention, and handling procedures for anthrax [7]. Agents commonly cited to inactivate anthrax spores include formaldehyde, hypochlorite solutions, chlorine dioxide, and radiation [8]. However, most of these agents are harmful to humans, limiting their use in public environments. Therefore, a safer disinfection technique that can exert a sustainable antimicrobial effect in human living environments is highly desirable.

Photocatalytic titanium dioxide (TiO_2_) substrates have been demonstrated to eliminate organic compounds and to function as disinfectants [9]. On stimulation by ultraviolet (UV) light irradiation, the photon energy excites valance electrons and generates pairs of electrons and holes (electron vacancy in the valence band) that diffuse and become trapped on the TiO_2_ surfaces. These excited electrons and holes have strong reducing and oxidizing activities and react with atmospheric water and oxygen to yield reactive oxygen species (ROS) such as hydrogen peroxide (H_2_O_2_), hydroxyl radicals (•OH), and superoxide anions (O_2_^−^) [10], which are extremely reactive on contact with organic compounds, and have been shown to operate in concert to attack polyunsaturated phospholipids and DNA in bacteria [9,11]. The oxidation of bacterial cell components such as lipids and DNA might therefore result in subsequent bacterial cell death [9]. Consequently, the TiO_2_ photocatalytic process is a conceptually feasible disinfectant technology.

The TiO_2_ photocatalyst, however, is effective only on irradiation with UV light at the necessary levels, which can induce severe damage to human eyes and skin [12,13,14,15]. This greatly restricts the potential applications of the photocatalyst for use in human living environments. To solve this problem, impurity doping of TiO_2_ with different elements has been used, including carbon, sulfur, nitrogen, and silver, resulting in excitation wavelength shifts from the UV to visible-light [16,17,18,19,20,21,22,23,24,25]. Simultaneously, the proper amount of impurity doping of TiO_2_ may also reduce the recombination rates of electron and hole pairs. Previously, we reported visible-light-responsive photocatalyst (VLRP) films, which offered a complementary and possibly alternative approach for meeting this need to control the spread of anthrax [24]. However, these VLRP films must be precoated on the surfaces of particular objects, whereas photocatalytic NPs do not, and as such may have broader applications. To solve this problem, the anti-anthrax properties of VLRP carbon-containing titanium dioxide [TiO_2_(C)] nanoparticles [TiO_2_(C) NPs; C200 NPs] [17] were evaluated in this study. The visible-light-responsive photocatalytic activity of C200 NPs has been respectively validated by degradation of methylene blue in liquid phase, oxidation of NO in gas phase, and sterilization in these works under visible light illumination [17,19,26,27,28]. The existence of carbonaceous species on TiO_2_ surface was analyzed by X-ray photoelectron spectroscopy (XPS) and diffuse reflectance infrared Fourier transform spectra. The effect of carbonaceous species on physical properties was observed on UV-visible absorption spectra, photoluminescence spectroscopy, and Raman spectroscopy as shown in our previous works [26,27,28]. In addition, we have further demonstrated that C200 NPs exert superior *Escherichia coli* killing properties under visible light illumination when compared to anatase TiO_2_ NPs [17,19]. These results collectively suggested that the C200 NPs exhibit a photocatalytic property under visible light illumination. However, whether C200 NPs can eliminate spore-forming bacteria such as *Bacillus* species has remained uncertain. Therefore, the visible-light-responsive C200 NP-mediated anti-anthrax property was evaluated. The potential applications are discussed herein.

## 2. Results

### 2.1. Analyses of TiO_2_ NPs

**Figure 1:**
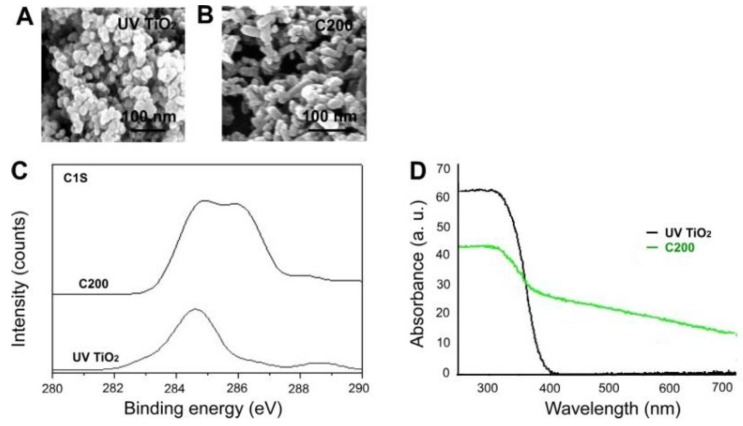
Scanning electron microscopy and ultraviolet-visible (UV-Vis) absorption spectrum analyses. Scanning electron microscopy (**A**,**B**), X-ray photoelectron spectroscopy (XPS) analysis for the 1s atomic orbital of carbon (**C**) and UV-Vis absorption spectra (**D**) of UV100 TiO_2_ and C200 NPs used in this study. The C200 sample absorbed light extending into the visible (>380 nm) region.

Detailed physical properties of UV-responsive pure TiO_2_ (TiO_2_; UV100 TiO_2_) and carbon-containing TiO_2_ (C200) NPs have been characterized in our previous work [17,26,27]. In the present study, scanning electron microscopy and UV-Vis absorption analyses of the newly prepared C200 NPs were performed (Figure 1). We found that both TiO_2_ and C200 displayed nanoscale structures (Figure 1A,B), and that an increased content of carbon (Figure 1C) and C200 displayed considerable redshift absorbance compared with TiO_2_ NPs (Figure 1D), indicating absorbance in the visible light range (wavelength > 380 nm). The UV-Visible diffuse reflectance spectra were converted by instrument software to absorbance values, *F*(*R*), based on the Kubelka-Munk theory. In the C200 sample, a sharp edge extending to approximately 438 nm and corresponding to a band gap of approximately 2.83 eV was observed, as indicated in one of our previous reports [27].

### 2.2. Dose-Dependent and Kinetic Analyses of Photocatalytic Inactivation of B. Subtilis

**Figure 2:**
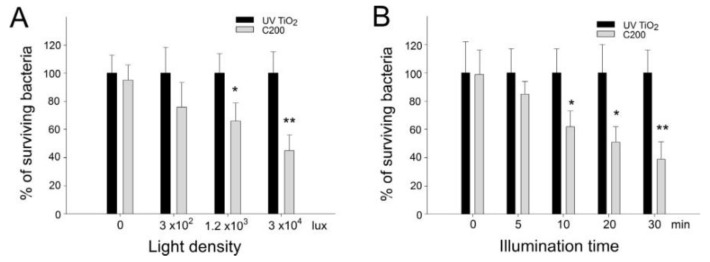
Dose-dependent and kinetic analyses of bactericidal activity of C200 NPs against *B. subtilis*. Dose-dependent (**A**) and kinetic (**B**) analyses of the bactericidal activity of UV100 TiO_2_ and C200 NPs against *B. subtilis* after visible-light illumination. Illumination was carried out either at different light densities (at distances of 5 cm, 10 cm and 15 cm with respective illumination intensities of 3 × 10^4^, 1.2 × 10^3^ and 3 × 10^2^ lux) for 30 min (**A**) or at a light density of 3 × 10^4^ lux (90 mW/cm^2^) for different periods (**B**). Under each illumination condition, the surviving bacteria in the UV100 TiO_2_ groups were normalized to 100%. * *P* < 0.05 and ** *P* < 0.01 compared with the respective UV100 TiO_2_ groups. *n* = 6, three experiments with two replicates.

The antibacterial properties of C200 NPs have been demonstrated [17]; however, whether C200 can also functionally eliminate spore-forming bacteria such as *B. anthracis* and *B. subtilis* remains to be further elucidated. Because *B. anthracis* is hazardous to humans, before analysis using *B. anthracis*, we employed *B. subtilis* as a surrogate. To obtain dose-dependent and kinetic data for *B. subtilis* with C200 NPs, we further analyzed the effects of illumination by visible light at various time points and at various distances (5 cm, 15 cm, and with different illumination intensities of 3 × 10^4^ and 5 × 10^2^ lux (lumen/m^2^); Figure 2A). The results indicated that C200 substrates can inactivate *B. subtilis* in half an hour when exposed to various degrees of illumination by visible light (Figure 2B). The bacteria-killing efficiency in the C200 groups was significantly higher than in the respective UV100 TiO_2_ groups (Figure 2A,B; ** *P* < 0.01, * *P* < 0.05).

### 2.3. Antibacterial and Antispore Activities of C200 against Bacillus Species

**Figure 3:**
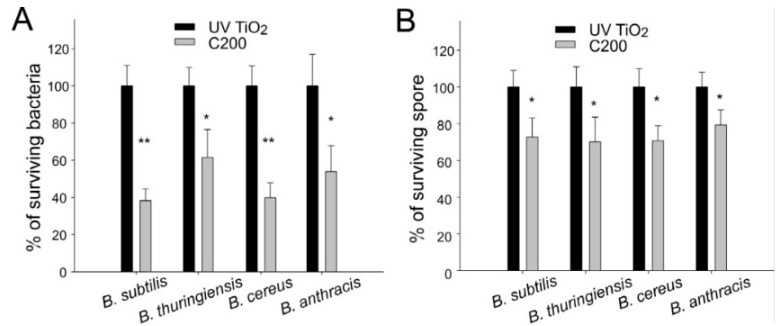
Antibacterial properties of C200 NPs against vegetative bacteria and spores of *Bacillus* species. Bacteria *B. subtilis*, *B. thuringiensis*, *B. cereus*, and *B. anthracis* were photocatalyzed using UV100 TiO_2_ and C200 NPs, respectively. All vegetative bacteria (**A**) or spores (**B**) in the UV100 TiO_2_ groups were normalized to 100%. The relative percentages of surviving pathogens in the C200 groups are shown. The illumination intensity was 3 × 10^4^ lux (90 mW/cm^2^), and the reaction time was 30 min. * *P* < 0.05 and ** *P* < 0.01 compared with respective UV100 TiO_2_ groups. *n* = 6, three experiments with two replicates.

Photocatalyst-mediated killing was performed to determine the antibacterial and antispore effects of photocatalysis on *B. cereus*, *B. thuringiensis*, and *B. anthracis*. Compared with UV100 TiO_2_, we found that C200 NPs exhibited higher visible-light-responsive antibacterial properties to kill *B. cereus, B. thuringiensis*, and *B. anthracis* vegetative bacteria (Figure 3A, * *P* < 0.05, ** *P* < 0.01). Consistent with the antibacterial experiment, in the spore analysis, C200 also exhibited a superior visible-light-responsive antispore activity compared with the UV100 TiO_2_ NPs, although this activity was less efficient (20%–30% killing/inactivation) (Figure 3B, * *P* < 0.05) compared with the results obtained in vegetative bacteria experiments (Figure 3A).

### 2.4. Photocatalytic Inactivation of Anthrax LT by C200 NPs

**Figure 4:**
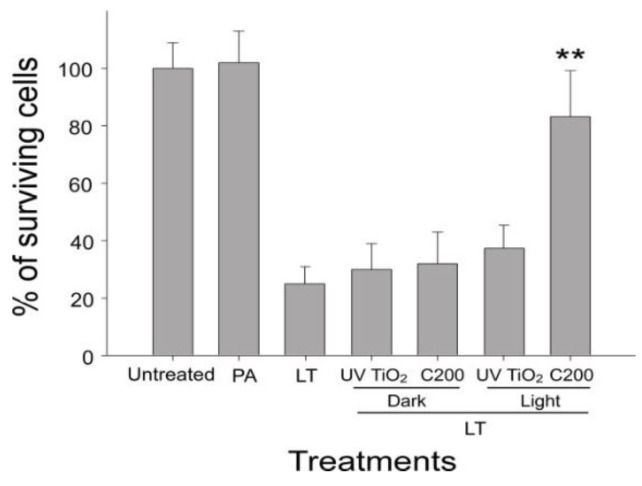
Visible-light-responsive C200 NP-mediated inactivation of lethal toxin (LT). Macrophage J774A.1 cells were treated with LT with or without UV100 TiO_2_ and C200 photocatalysis for 3 h, and surviving cells of untreated groups were adjusted to 100%. Columns designated UV TiO_2_ or C200 represent that LT was pretreated with photocatalysis by using UV100 TiO_2_ or C200 NPs, respectively, before being treated with J774A.1 cells. ** *P* < 0.01, compared with all other groups treated with LT (with or without additional treatments). *n* = 6, three experiments with two replicates.

Anthrax lethal toxin (LT), which is composed of two protein components—a protective antigen (PA; cell receptor binding) and a lethal factor (LF; metalloprotease of cellular mitogen-activated protein kinase kinase (MAPKKs, MEKs))—is one of the major virulence factors of *B. anthracis* [29]. Anthrax LT can be detected in culture media and spores of *B. anthracis*. Treatments of LT can lead to the death of macrophage cells in vitro [30] and lead to mortality in rodents [24,31,32,33,34,35]. To investigate whether photocatalysis can inactivate the protein components and detoxicate the anthrax toxin, LT was subjected to visible-light-activated photocatalysis on UV100 TiO_2_ and C200 NPs. As expected, C200 NP-mediated photocatalysis markedly reduced the potency of LT to induce cell death of macrophage J774A.1 cells, when compared with UV100 TiO_2_ groups (Figure 4). These results suggested that treatment with visible-light-responsive C200-mediated photocatalysis of particular objects not only eliminates the bacteria but also reduces the toxicity of LT.

### 2.5. In Vitro Phagocytic Clearance Analysis

**Figure 5:**
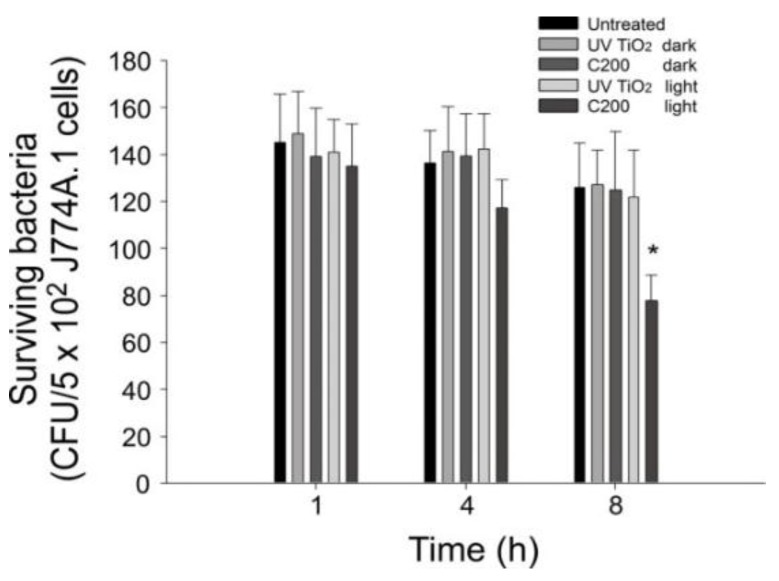
Surviving *B. subtilis* after clearance by macrophages. *B. subtilis* was treated with J774A.1 macrophage cells (multiplicity of infection (MOI): 0.1 bacteria/cell). Levels of surviving bacteria (colony-forming unit; CFU) harvested from macrophage cell lysate are shown. Columns designated UV TiO_2_ and C200 represent that anthrax spores were pretreated with photocatalysis by using UV100 TiO_2_ and C200 NPs, respectively. * *P* < 0.05, compared with all other groups under the 8 h treatment condition. *n* = 6, three experiments with two replicates.

Our previous reports suggest that VLRP-mediated photocatalysis can injure bacteria [17,18,19,24,25] and lead to faster clearance of bacteria by phagocytes [24]. To investigate whether photocatalysis might injure the bacterium and make it more vulnerable to phagocyte-mediated killing, *B. subtilis* was used as a surrogate for *B. anthracis* to treat macrophage J774A.1 cells. To avoid a biased condition, we applied a subbacterial killing condition (3 × 10^4^ for 5 min), under which bacterial survival could not be considerably suppressed (Figure 2B). The analysis results revealed that *B. subtilis* in groups without antibacterial photocatalytic properties (i.e., UV100 TiO_2_ groups and C200 dark groups; Figure 2) tended to have a low reduction rate (killing by macrophages) after being engulfed by the phagocytes (Figure 5). By contrast, the levels of surviving bacteria of macrophage-engulfed *B. subtilis* were markedly suppressed 8 h after ingestion, only in C200 groups with visible-light illumination (Figure 5, C200 light group). These results suggested that the C200-mediated photocatalysis also induced damage in bacterial cells that could be repaired after plating (Figure 2B, 5 min group, not significant reduction), but resulted in a relatively vulnerable phenotype when encountering phagocytes (Figure 5, C200 light 8 h group). This suggested that, although the bacteria may not efficiently be killed when a subantibacterial dose of photocatalysis is applied, the bacteria that survive the photocatalysis are still more easily secured and eliminated by phagocytes in the immune system. In other words, the C200-mediated photocatalysis exhibits a protective effect against contaminated bacteria even under subbacterial killing conditions.

## 3. Discussion

Disinfection is a vital method for controlling numerous pathogens in the sterilization of critical instruments, water treatment, food production, and hospital environments. Traditional chemical-based disinfectants, such as alcohols, aldehydes, iodine, phenols, and chlorine, have been used for centuries in environmental cleaning [18]. Among these, agents and methods such as formaldehyde, hypochlorite solutions, chlorine dioxide, and radiation have been used to inactivate anthrax spores [8]. Although these methods may be effective, they have drawbacks. Many of these disinfectants are volatile, and their byproducts can be toxic and carcinogenic to humans. The establishment and development of novel anti-anthrax strategies are necessary.

Compared with chemical disinfectants, the TiO_2_ photocatalyst is safe, nontoxic, and produces no hazardous byproducts [36,37]. Utilization of the excellent photocatalytic antibacterial effect of TiO_2_ appears to be a conceptually feasible technology for solving the aforementioned problem of chemical disinfectants. However, traditional antibacterial TiO_2_ photocatalysts are activated by UV irradiation, which is hazardous to humans [12,13,18]. The UV-responsive TiO_2_ photocatalysts are therefore unsuitable for application in indoor environments. By contrast, visible-light-activated TiO_2_ photocatalytic NPs exhibit at least four advantages. First, compared with UV-responsive materials, visible-light-activated TiO_2_ photocatalysts offer potential for use as a disinfectant in human living environments such as indoor spaces and public areas. Second, because TiO_2_ is a chemically stable and inert material, it can continuously exert antimicrobial action when illuminated by light. Third, the bactericidal activity can be switched on and off or modulated by controlling the light intensity. Fourth, the transportability of NPs offers greater adjustability. Because of these advantages, visible-light-activated photocatalytic NPs might be used complementarily with existing disinfection technologies against anthrax.

Emerging nanomaterials and particular NPs have been implicated as having tremendous potential applications in environmental and disinfection use [38]. We previously demonstrated that photocatalytic films can eliminate vegetative bacteria and spores of anthrax [24]. However, compared with nanostructured films, which have to be precoated at the surfaces of particular objects, NPs frequently show superior portability [39]. For example, VLRP NPs can theoretically be easier to spread into a dead corner of spore-contamination-suspected objects, houses, territorial waters, and even aerosol spaces, when compared with VLRP films. Therefore, in the present study, visible-light-responsive C200 NPs were used. For toxicity consideration, previous studies have shown that TiO_2_ NPs are generally considered safe for skin contact or even ingestion by healthy animals [40,41,42]. However, inhalation of TiO_2_ NPs can be toxic to the lungs [42]. As a result, in a real setting, aerosol TiO_2_ NPs should be avoided unless the nearby persons are provided with appropriate certified masks (e.g., N95) and/or air cleaning and filtering equipment. A pioneering work demonstrated that supplements with various types of NPs, including TiO_2_ NPs, may reduce the growth of *Escherichia coli* and *B. anthracis* in bacterial culture [43]; however, it showed that the growth retardation effect was due to the presence of excessive NPs in the culture medium rather than bacterial killing. In addition, the photocatalytic effect of TiO_2_ NPs on *B. anthracis* has not yet been reported. Therefore, to determine a more efficient way to eliminate the contaminated pathogenic bacteria, relevant verifications that involve using photocatalytic NPs are necessary.

Carbon-containing titanium dioxide C200 NPs exhibit considerably superior photocatalytic properties under visible-light illumination compared with UV100 TiO_2_ NPs [17,19,24,26,27,28]. However, whether C200 NPs can eliminate spore-forming bacteria such as *Bacillus* species has remained elusive. Therefore, in the present study, we demonstrated that visible-light-responsive C200 NP-mediated photocatalysis can eliminate considerable levels of both vegetative bacteria and spores of Bacillus species, including *B. subtilis, B. cereus, B. thuringiensis*, and *B. anthracis*. The analysis data suggested that C200 NPs are useful for eliminating contamination of these spore-forming bacteria. Notably, in agreement with our previous findings [24], our data suggest that C200-mediated photocatalysis somehow injures the bacteria even under suboptimal antibacterial conditions, thus leading to faster clearance of the photocatalyzed bacteria by phagocytes (Figure 5). This suggests that C200 can still exert anti-anthrax properties even at a subantibacterial level.

## 4. Materials and Methods 

### 4.1. Preparation of Photocatalysts

Visible-light-responsive carbon-containing mixed phase TiO_2_ nanoparticles [TiO_2_(C) NPs; C200 NPs] were prepared using a modified sol-gel method as previously described [17,26,27]. A measured quantity of tetrabutyl orthotitanate (50 mmol) was slowly introduced into 90 mL of anhydrate ethanol and 20 mL of deionized (DI) water in a 250 mL flask. After complete dissolution, 4 mL of nitric acid was added to catalyze the hydrolysis and condensation reactions. The mixed solution was uniformly agitated at 500 rpm for 3 h, producing a precipitate of titanium hydroxide. After drying at 110 °C, the dried powder was calcined in air at 200 °C for 5 h and designated as C200 [17,24,26,27]. Details of preparing the production of C200, including structural properties and the sizes of primary particles, were reported in our earlier work [27]. The surface of C200 was found to contain unique anatase/rutile mixed crystalline phases that exhibit strong visible-light absorption and photocatalytic effects [22,23]. The carbonaceous species on TiO_2_(C) NPs exist in an amorphous form, as observed in Raman spectra [26,27,28]. One commercially available TiO_2_ NP (UV100, Sachtleben, Germany) that can exert photocatalytic properties only when illuminated by UV light was used for comparison. Because C200 samples often aggregate into larger clusters because of surface charges, in Van der Waals interactions, we dispersed the aggregates by using sonication (Transsonic Digital TP680DH, Elma Schmidbauer, Singen, Germany) before the bacteria-killing or bacteria-photocatalyst interaction experiments. The UV-Vis absorption spectra were recorded on a Hitachi 3300H spectrophotometer (Hitachi Taiwan, Taipei, Taiwan) [24,44]. Particle size and morphology were determined using a transmission electron microscope (Philips Tecnai F20 G2 FEI-TEM, Philips Taiwan, Taipei, Taiwan) and a scanning electron microscope (JEM-3010, JEOL, Tokyo, Japan) [17,21]. Material compositions were determined using X-ray photoelectron spectroscopy (Perkin Elmer SSI-M probe XPS system and S4800, Waltham, MA, USA).

### 4.2. Bacterial Strains and Culture

Bacterial culture and maintenance were conducted according to previously described methods [24]. *B. anthracis* (ATCC 14186) containing both pXO1 and pXO2 plasmids to express functional LT and edema toxin (ET) was grown on blood agar plates (BAPs) and maintained in brain–heart infusion broth (BHIB) (Sigma-Aldrich, St. Louis, MO, USA) using previously described methods [17,24,30,31,32,33,34,45,46,47]. *B. cereus* (ATCC 13061) and *B. thuringiensis* (ATCC 35646) were maintained and cultured in BAPs or BHIB at 30 °C, and *B. subtilis* (ATCC 39090) was maintained and cultured in trypticase soy agar or broth (Sigma-Aldrich, St. Louis, MO, USA) at 37 °C [24,48]. Bacterial strains were stored in a 50% culture medium and 50% glycerol solution at −80 °C before use. To reactivate bacteria from frozen stock, 25 µL of bacterial stock solution was transferred to a test tube containing 5 mL of a freshly prepared culture medium and then incubated at 30 °C or 37 °C under agitation overnight (16–18 h). Spores of *B. anthracis* were prepared as previously described [24,49,50]. Overnight BHIB cultures of *B. anthracis* were diluted to approximately 10^7^ colony-forming units (CFU)/mL in phosphate-buffered saline, and 0.1-mL aliquots were inoculated onto blood agar plates. The agar plates were incubated at 25–37 °C until 90%–99% phase-bright spores were observed using phase-contrast light microscopy. Spores were harvested and washed with cold sterile DI water as previously described [49] and stored in the freezer at −20 °C for up to 1 mol. The quality of spores was determined by two complementary criteria previously established for validating the presence of dormant spores [50]. The criteria consisted of evaluating (i) the absence of vegetative cells (rods) determined through microscopic examination as described, and (ii) the survival of spores in hydrochloric acid (2.5 N). Spore preparations of *B. subtilis*, *B. cereus*, and *B. thuringiensis* were conducted following the same protocol.

### 4.3. Photocatalytic Reaction and Detection of Viable Bacteria

In this study, bacterial concentrations were either determined using the standard plating method or inferred from optical density readings at 600 nm (OD600). For each Bacillus species, a factor for converting the OD600 values of the bacterial culture to concentration (CFU/mL) was calculated as follows. A fresh bacterial culture was diluted by factors of 10^−1^ to 10^−7^, and the OD600 of these dilutions was measured. The bacterial concentrations of these dilutions were determined using the standard plating method. To determine the bactericidal effects of the TiO_2_-related substrates, 200 µL of bacterial overnight culture was transferred into 5 mL of a culture medium and incubated at 37 °C until an OD600 of 0.3–0.6 (log phase) was reached. The bacterial concentrations were calculated using the previously determined conversion factor for the bacteria, and the cultures were diluted to 1 × 10^5^ CFU/mL with the culture medium. The bacterial cultures (2.5 × 10^4^ CFU) were mixed with the TiO_2_ or C200 NPs (200 µg/mL in 150 µL of normal saline) using a 200 µL pipetman and placed onto a 24-well cell culture dish. Photocatalytic reactions were carried out using an incandescent lamp (Classictone incandescent lamp, 60 W, Philips Taiwan, Taipei, Taiwan). A light meter (model LX-102, Lutron Electronic Enterprises, Taipei, Taiwan) was used to record the illumination density. In the dose-dependent experiments, illumination was carried out for 5 min at distances of 5, 10 and 15 cm from the lamp, corresponding to illumination densities of 3 × 10^4^, 1.2 × 10^3^ and 3 × 10^2^ lux (lumen/m^2^) (equivalent to 90, 30 and 10 mW/cm^2^), respectively. In the kinetic analysis experiments, illumination was carried out for 1, 5, 10, 20 and 30 min at a distance of 5 cm, corresponding to an illumination density of 3 × 10^4^ lux (90 mW/cm^2^). Unless specified, illumination was carried out in a 4 °C cold room to prevent overheating of the photocatalyst-containing solution. After illumination, the levels of surviving bacteria were determined using a standard plating method immediately after bacterial collection. In the spore experiments, 1 × 10^4^ CFU (1 × 10^5^ CFU/mL in 100 µL) was used, and the procedures followed the same protocols as those in the live bacteria experiments.

### 4.4. Cytotoxicity Analysis

Cytotoxicity of anthrax LT was measured following previously described methods [30,45]. Anthrax LT is composed of two protein components, a PA (cell receptor-binding) and an LF (metalloprotease). The PA and LF were purified from *B. anthracis* (ATCC 14186) culture supernatants, as previously described [24,30,31,32,33,34,45,47]. The culture supernatants were filter-sterilized by being passed through a 0.22-mm filter (Millipore, Bedford, MA, USA) and concentrated using the Minitan Ultrafiltration System (Millipore, Bedford, MA, USA). Protease inhibitor phenylmethylsulfonyl fluoride 0.1 mM (Sigma-Aldrich, St. Louis, MO, USA) [51,52] was added to prevent toxin degradation. Ammonium sulfate was added to 75% to precipitate the protein; and the protein was collected and suspended in 20 mM tris(hydroxymethyl)aminomethane HCl (Tris-HCl) pH 8.0, and extensively dialyzed against the same buffer. The purification was further performed using fast protein liquid chromatography Mono Q anion exchange (Pharmacia, Piscataway, NJ, USA) with a 20 mM Tris-HCl pH 8.0 buffer and linear 0–400 mM NaCl gradient elution over 40 min. The PA was eluted at 130–140 mM NaCl, and the LF was eluted at 250–270 mM NaCl. Lipopolysaccharide (LPS) contamination was monitored using a Limulus Amoebocyte Lysate QCL-1000 kit (Lonza, Walkersville, MD, USA) [31,35,53]. Batches of purified LT with an LPS contamination level of less than 1 EU/mg of LT were used [31]. The LT solution was mixed with the TiO_2_ or C200 nanoparticles (200 µg/mL in 150 µL of normal saline) by using a 200 µL pipetman and placed onto a 24-well cell culture dish. After inactivation in visible-light illumination for 30 min (3 × 10^4^ lux; 90 mW/cm^2^), the LT (LF:PA = 1:5) was ready for use. Cytotoxic doses of LT (10 mg/L) [30], with or without pretreatment of photocatalytic inactivation using pure TiO_2_ and C200 NPs, was used to treat mouse macrophage J774A.1 cells. Three hours after the LT treatments, the cell viability of J774A.1 cells was measured using a WST-1 kit (Roche, Mannheim, Germany) [30,53,54], following manufacturer instructions.

### 4.5. Phagocytosis Analysis

*B. subtilis* was dissolved in normal saline (100 µL, 1 × 10^5^ CFU/mL) and then incubated with TiO_2_ and C200 NPs. The bacterial cultures (2.5 × 10^4^ CFU) were mixed with TiO_2_ or C200 NPs (200 µg/mL in 150 µL of normal saline) using a 200 µL pipetman and placed onto a 24-well cell culture dish. The bacterium–photocatalyst mixtures were then illuminated with visible light (Classictone incandescent lamp, 60 W, Philips; 90 mW/cm^2^; lamp target distance 10 cm) for 30 min in 24-well plates. After illumination, the bacteria-containing solutions (85 µL) were recovered from the 24-well plates. The bacterial solution was then added into one well of a six-well cell culture dish containing confluent murine macrophage J774A.1 cells (1 × 10^6^ cells/well; MOI: 0.1 bacteria/cell). After phagocytosis was carried out for 1 h, the culture medium was removed, and 200 µL of a cell lysis buffer (100 mM Tris-HCl [pH 7.4], 10 mM MgCl_2_, 100 mM NaCl, 0.2% sucrose, 0.5% Triton X-100) modified from that described in previous literature [55,56] was then added to release the cell-engulfed or cell-bound bacteria. A serum-free and antibiotic-free cell culture medium (Dulbecco’s modified Eagle’s medium) was used in this analysis.

### 4.6. Statistical Analyses

The means, standard deviations, and statistics for the quantifiable data were calculated using Microsoft Office Excel 2003 (Microsoft, Redmond, WA, USA), SigmaPlot 10 (Systat Software, San Jose, CA, USA), and SPSS Statistics for Windows, Version 19.0 (IBM, Armonk, NY, USA). Significance of the data was examined using one-way ANOVA followed by the post hoc Bonferroni-corrected test. The probability of type 1 error α = 0.05 was recognized to be the threshold of statistical significance.

## 5. Conclusions 

In the present study, we demonstrated that C200 NPs are conceptually feasible nanomaterials for eliminating the vegetative cells and spores of *B. anthracis*. In particular, C200-mediated photocatalysis exhibits a protective effect against the bacteria, even under suboptimal conditions. Our results collectively suggest that C200 NPs and the extended concepts and technologies described in this report are useful for the development of new strategies against anthrax.

## Abbreviations

The following abbreviations are used in this manuscript:

TiO_2_	titanium dioxide
TiO_2_(C)	carbon-containing TiO_2_
VLRP	visible-light-responsive photocatalyst
NPs	nanoparticles
UV	ultraviolet
ROS	reactive oxygen species
H_2_O_2_	hydrogen peroxide
•OH	hydroxyl radicals
O_2_^−^	superoxide anions
LT	anthrax lethal toxin
PA	anthrax protective antigen
LF	anthrax lethal factor
ET	anthrax edema toxin
MOI	multiplicity of infection
CFU	colony-forming units
LPS	lipopolysaccharide
